# Two years’ study of Tetanus cases in a Paediatric Intensive Care Unit

**DOI:** 10.12669/pjms.323.9165

**Published:** 2016

**Authors:** Faizia Naseem, Imtiaz Ahmad Mahar, Fehmina Arif

**Affiliations:** 1 Faizia Naseem, MBBS, DCH, MCPS, FCPS. Senior W.M.O, Paediatric Unit-II, Civil Hospital, Karachi, Pakistan; 2 Imtiaz Ahmad Mahar, MBBS, DCH. Senior M.O, PICU, Civil Hospital, Karachi, Pakistan; 3 Fehmina Arif, MBBS, DCH, FCPS. Professor, Department of Paediatrics, Dow Medical College, Dow University of Health Sciences, Karachi. Pakistan. Civil Hospital, Karachi, Pakistan

**Keywords:** Tetanus, Outcome, Burden

## Abstract

**Objective:**

To study the demographic and clinical features, outcome, complications and treatment cost of tetanus patients admitted in Paediatirc Intensive Care Unit (PICU) of Civil Hospital Karachi (CHK).

**Methods:**

It is a descriptive observational study conducted at Civil Hospital Karachi from July 2013 to June 2015. Patients of tetanus admitted in PICU during the study period were enrolled. Data was collected from the file records of patients and included the demographic profile, clinical presentation, grade of severity, length of stay, complications and outcome. It also included the cost of treatment. Descriptive statistics were applied to describe the results.

**Results:**

During the study period, 23 cases of tetanus were admitted in P.I.C.U. twelve were male and 11 female. Majority of cases (13) belonged to age group 2-6 years. Seventeen cases were unvaccinated and 6 had received only BCG & OPV. None was appropriately vaccinated for age.

There were 9 cases of post injury tetanus, 6 of them were males, 5 cases of otogenic tetanus and 9 cases had no clinically identifiable portal of entry. Eleven cases belonged to grade III severity of Ablett classification and 6 had grade IV severity. Mortality in our case series was 26%. Autonomic instability was seen in 17 patients and all of them needed ionotropic support. The estimated cost of per day treatment of a tetanus patient with mechanical ventilation was approximatly 31, 979/Pak Rs and without mechanical ventilation was 20,000/Pak Rs.

**Conclusion:**

Tetanus is an entirely preventable disease with a high mortality. Treatment is very costly as compared to vaccination which is free of cost. Complete vaccination and proper wound care is the only option to reduce the ongoing burden of tetanus.

## INTRODUCTION

Tetanus is an acute, potentially fatal disease caused by Clostridium Tetani which produces a powerful neurotoxin tetanospasmin which affects the central nervous system.^1^ Although tetanus is almost entirely preventable through immunization, the burden of disease is large world wide. The global incidence of tetanus is still estimated at one million cases annually, with a case fatality ratio ranging form 20% to over 50%.^2^

Most cases of tetanus follow an acute penetrating skin injury. The injury may be major but often is trivial, so that medical attention is often not sought.^2^ Tetanus is also associated with ulcers, burns, gangrene, snake bite, septic abortion, child birth, otitis media, intramuscular/intravenous injections and surgery.^3^

Outbreaks of tetanus related to injuries associated with natural disasters such as earth quakes and tsunamis have been documented.^4^-^6^ Wherever the immunization programs are in place, the incidence of tetanus declines and the age distribution of case-patients shifts to reflect under immunization.^7^ So due to lack of proper immunization programs, tetanus is still endemic in many developing countries.^2^

In developed countries, the widespread use of tetanus toxoid for active immnization, improved wound care management and the use of tetanus immunoglobulin (TIG) for post exposure prophylaxis and for treatment, have contributed greatly to decrease the incidence of tetanus as such and its morbidity and mortality as well.^8^

This study was carried out to share our two years experience of managing tetanus patients, focusing on the demographic profile, clinical presentation, severity, length of stay, complications, outcome and the management protocol used in our PICU. We also wanted to point out the economic burden due to lengthy and costly treatment of tetanus which can be easily prevented by vaccination, the absence of which will let the burden continue.

## METHODS

Children aged one month to 12 years admitted in P.I.C.U (as per hospital admission policy) with the clinical diagnosis compatible with tetanus were enrolled. Data was collected from the records of patients. Data included the demographic profile, clinical presentation, grade of severity, length of ICU stay, complications and outcome. It also included the cost of treatment. Descriptive statistics were applied to describe the results.

### Management Protocol

Management of tetanus emphasises upon wound care, neurtralization of the toxin, antibiotic therapy, supportive measures including good nursing care with control of convulsions and completion of active immunisation.

Upon arrival the patients were assessed thoroughly for their vitals, respiratory status, grade of severity, portal of entry and wound status (if present) along with systemic examination.

All the patients were kept in isolation to avoid light, noise and other disturbances. Strict aseptic measures were taken throughout. Wound toilet was carried out and all the patients were given human T.I.G in a dose ranging from 1500-3000 i.u. They were also given 0.5ml tetanus toxoid as part of their active immunization. All the patients were started on diazepam 5-10mg/kg/day, 50% of dose being given i/v, and 50% orally in four divided doses. Few of them required a dose of 20mg/kg/day q 6 hourly.

Patients were given phenobarbitone 20mg/kg as a loading dose i/v and 5 mg/kg /day as maintenance dose initially i/v and later via NG tube. They were also given chlorpromazine 0.5-1 mg/kg/dose every 6 hourly, initially i/v and later orally. All the patients were also started on injectable magnesium sulphate in a loading dose of 70-75mg/kg and maintained on 20-25 mg/kg/dose q 8 hourly for an average duration of one week. It is used in combination with benzodiazepins to control spasms and autonomic dysfuction. It is a presynaptic neuromuscular blocker and reduces catecholamine release from nerves and the adrenal medulla and reduces responsiveness to released catecholamines.^9^

Those patients who needed ventilator were given inj. Midazolam 0.1mg/kg/hour and atracurium 1mg/kg/hour. In such cases, the total dose of diazepam was reduced to 50%.

All the patients were given I/V antibiotics, metronidazole for 10-14 days and if they had assoicated otitis media or other focus of infection then 3^rd^ generation cephalosporins or benzyl penicillins were added.

Seventeen patients also required inotropic support in the form of I/V dopamine and / or epinephrine infusion. All the patients were also given PPIs, proton pump inhibitors (omeprazole) and prokinetic agent domperidone. Nasogastric feeding was started with in 24 hours after inital stabilization. It was gradually built up provided there was no issue of gastric residue. Patients were also provided with physiotherapy after initial phase of stabilizaion.

Muscle relaxants were gradually tapered to minimum required doses (to prevent stiffness and spasms) and advised to continue for atleast 3-4 months or longer since prolonged convalescence with residual muscle rigidity is seen for several months in tetanus.^1^

Parents were counselled about the importance of vaccination and they were provided with a schedule to complete active immunization of their child and his/her siblings as well (if unvaccinated).

## RESULTS

There were 23 cases of tetanus, admitted to P.I.C.U during the study period July 2013 till June 2015. Twelve were male and 11 female. Majority of cases belonged to age group between two to 6 years. None of the cases was seen under two years age.

Regarding the vaccination status, 17 cases were unvaccinated, six were partially vaccinated (only BCG & OPV at birth) and none was appropriately vaccinated for age. Table-I

**Table-I:** Age, sex and vaccination status of tetanus patients (n=23).

	*No.*	*%*
*Age:*		
<2 yrs	0	
2-6 yrs	13	56.50
>6 yrs	10	43.47
*Sex:*		
Male	12	52.17
Female	11	47.82
*Vaccination Status:*		
Unvaccinated	17	
Partially vaccinated	6	73.91
Appropriately		26.08
Vaccinated for age	0	

Mode of acquiring tetanus is shown in Table-II. Otogenic tetanus was exclusively seen in 2-6 year age group. Among 9 cases of post injury /trauma tetanus, 7 had trauma to lower limbs. In remaining 9 cases, there was no clinically identifiable portal of entry.

**Table-II:** Mode of infection in tetanus case (n=23).

	*< 2 years old n=0*	*2-6 years n=13*	*>6 years -12 years (n=10)*
Otogenic	0	5	0
Post injury	0	4	5
Unknown	0	4	5

The average incubation period (IP) in post trauma cases was seven days. Determining the IP in otogenic cases was not possible because of chronicity of most otorrheas. Body stiffness/spasm, locked jaw and dysphagia were the three commonest presenting complaints in our series of tetanus cases.

According to the grade of severity (Ablett classification), majority of our patients i.e 11 (47.82%) belonged to grade III. Six patients had grade IV and none of the patients had grade I. Sensorium was not altered in majority 19 (82.60%), although patients were lethargic at presentation. Four patients had altered GCS ranging 12-8. Regarding the outcome, 17 cases were shifted to ward and 6 cases (of grade IV severity) expired.

All the severe cases had a period of onset of less than 48 hours. Regarding the length of stay, majority of our cases (15 out of 23) stayed for more than 3 weeks. Three of them stayed for more than 6 weeks. Table-III.

**Table-III:** Ablett grade of severity, out come and length of stay (LOS) (n=23).

	*No*	%
*Grade of Severity:*		
I	0	
II	6	26.08
III	11	47.82
IV	6	26.08
*Outcome:*		
Survived	17	73.91
Expired	6	26.08
*Length of ICU stay*:		
< than 2 days (48 hrs)	2	8.69
3-10 days	2	8.69
11-20 days	4	17.39
21-30 days	12	52.17
>31 days	3	13.04

Fifteen patients under went intubation and mechanical ventilation for an average duration of 10 days (8-12 days). Of these 15 patients, 5 were of grade IV severity and 10 were of grade III severity. Two patients had tracheostomy which was subsequently closed. Table- IV

**Table-IV:** Mechanical ventilation, autonomic instability, use of inotropes and packed cell transfusion in tetanus cases n=23.

	*No*	*%*
*Autonomic instability:*		
Present	17	73.91
Mechanical	15	65.21
Ventilation		
Tracheostomy	2	8.69
Packed cell transfusion	15	65.21
Inotropes required	17	73.91

During P.I.C.U stay, 15 cases required packed cell transfusion to build up Hb to 10gm percent. Eighty five percent of these had microcytic anemia of iron deficiency. Autonomic instability was seen in 17 patients, including all 6 cases of grade IV severity and 11 cases of grade III. All these needed inotropic support. Some patients had associated comorbids in the form of pneumonia (1), ASD (1), ascariasis (1), and measles encephalitis (1).

### Complications

Following complications were documented in our patients. LRTI (lower respiratory tract infection) (5), hypotension (17), AKI (according to rifle criteria) (15), sepsis (10), paralytic ileus/constipation (20), and one boy developed relapse of nephrotic syndrome. One patient deveoped toxic shock syndrome and expired. Hypoxic brain injury (radiologically proven) resulting in neurological deficits was observed in three patients. They were put on antiepileptics due to persistence of convulsions.

## DISCUSSION

In this retrospective, descriptive study, a high incidence of tetanus was found in the 2-6 year age group, which is in accordance with other regional studies.^10^ Male predominance was seen in post trauma cases (7 out of 9, 77.77%), because the boys are more involved in outdoor activites as compared to girls.^2^

Regarding the portal of entry, Otogenic route was exclusively confined to the 2-6 year age group (n=5, 21.7%) as otitis media is common in this age group. Introduction of unlcean fingers and contaminated objects into the ears is also common in this age.^10^

Regarding the site of trauma, majority (6 out of 9, 66.66%) had injury to lower limbs (toe, sole, shin) which is also suppported by other studies.^10^,^11^ One of our patient deveoped tetanus after dog bite.^1^

Unlike other diseases, tetanus is entirely preventable by immunization,^12^ A five dose regimen of tetanus toxoid provides adequate immunity. Routine tetanus booster vaccination is recommended for adolescents and adults, every 10 years.^13^

It was noted that none of our patients was vaccinated for tetanus, which is very alarming because despite the continuous efforts of health sector, vaccination status of our children in general is falling and currently it is approx 54% (for all vaccine preventable diseases).^14^ Since Pakistan is an agricultural state and the disease is common where soil is cultivated, in rural areas, warm climates and among males, so being a tetanus prone country, vaccination against tetanus is imperitive for our children.^2^

Similar to other studies, locked jaw/trismus, body stiffness/spasm and dysphagia were the commonest presenting complaints in our series.^2^,^6^,^10^,^11^ Hence a high index of suspicion for tetanus should be excercised whenever patients present with any of these symptoms irrespective of history of trauma. 2

There are many scoring systems for grading the severity of tetanus. We adopted the Ablett classification.^15^ According to it, we had majority of severe cases i.e 11 cases of grade III. Six cases belonged to grade IV (who all expired) none was found in grade I (Table III).

Tetanus patients usually require lengthy ICU stay and number of drugs. As proved by our case series and supported by previous studies, the longer the duration of hospital stay, the more favorable the outcome.^11^

Autonomic dysfunction is common in tetanus.^16^,^17^,^18^ It usually starts by the end of 1^st^ week of illness and persists for 1-2 weeks. It is due to the effect of tetanus toxin on the brain stem and autonomic interneurons. Although it is mainly due to paroxysmal increases in sympathetic activity resulting in hypertension, tachycardia and pyrexia, at times there is parasympathetic over activity resulting in hypotension and bradycardia added by heavy doses of benzodiazepines and other sedatives.^19^ It was seen in 17 of our patients who all needed inotropic support to maintain BP between 50^th^ and 90^th^ centile

Both factors lead to increase the cost of treatment. As shown in table III, majority (15 out of 23) stayed for more than three weeks. On the other hand, the total cost of tetanus vaccination series is approximately 300-400 Pak rupees per person. Hence we have no option other than proper vaccination. It is said that prevention is better than cure, for tetanus; it is easier and cheaper also. Although elective tracheostomy has been emphasized for moderate to severe cases, this was not possible for us due to limited resources.

Fifteen of our patients needed mechanical ventilation. One patient had tracheostomy straight away while other was subjected to post extubation tracheostomy due to persistant stridor. Regarding the outcome, tetanus has a mortality rate ranging between 20 to over 50% as mentioned in various studies.^2^,^6^,^10^,^17^,^20^,21 Mortality was 26% in our case series. These were cases with grade IV severity, having severe autonomic instability and respiratory compromise needing mechanical ventilation.

We have calculated the per day treatment cost of a tetanus patient, which is approx 20,000 pak rupee without ventilator and approx 31,979 pak rupee with ventilator. If a patient stays for three weeks, (which is the usual case) the figure goes in lacs for complete treatment. This represents a drain on existing intensive care funds since on the other hand vaccination for tetanus can be completed in 300-400 Pak rupees only (In private setups), where as it is provided free of cost by the government under EPI Programme.

## CONCLUSION

Complete vaccination and proper wound care is the only option to save our children from tetanus… a disease of high morbidity and mortality, and high treatment cost.
